# 我国东部饮用水源水中传统及新型全氟和多氟烷基物质的赋存特征及风险评估

**DOI:** 10.3724/SP.J.1123.2024.09004

**Published:** 2025-08-08

**Authors:** Jianglong LIU, Bing ZHANG, Wen GU, Deling FAN, Lei WANG, Heyun FU, Ningbo GENG, Shuai SUN

**Affiliations:** 1.生态环境部南京环境科学研究所，江苏 南京 210042; 1. Nanjing Institute of Environmental Sciences，Ministry of Ecology and Environment of the People’s Republic of China，Nanjing 210042，China; 2.南京大学环境学院，江苏 南京 210023; 2. School of Environment，Nanjing University，Nanjing 210023，China; 3.中国科学院大连化学物理研究所，辽宁 大连 116023; 3. Dalian Institute of Chemical Physics，Chinese Academy of Sciences，Dalian 116023，China

**Keywords:** 全氟和多氟烷基物质, 饮用水源水, 污染特征, 风险评估, per- and polyfluoroalkyl substances（PFAS）, drinking water sources, pollution characteristics, risk assessment

## Abstract

为探明我国东部地区饮用水源水中传统及新型全氟和多氟烷基物质（PFAS）的赋存情况及潜在风险，采用弱阴离子交换固相萃取-超高效液相色谱-三重四极杆质谱法（UPLC-MS/MS）分析了东部地区13处饮用水源地水样中50种传统及新型PFAS的污染水平和分布特征，并开展了生态及健康风险评估。结果表明，我国东部地区饮用水源水中共检出26种PFAS，总含量为80.0~282 ng/L，中位值为153 ng/L。东部地区饮用水源水中PFAS的检出水平相对较高，但全氟辛酸（PFOA）和全氟辛基磺酸（PFOS）的水平远低于我国生活饮用水卫生标准限值。饮用水源水中检出的PFAS以短链的全氟丁酸（PFBA）和全氟丁烷磺酸（PFBS）为主，二者分别占总PFAS含量的27.6%和20.8%；而长链PFAS（碳链长度>9）的占比低于2%。尽管检出水平较低，但在东部地区饮用水源水中已发现多种新型PFAS。相关性分析结果表明，饮用水源水中的PFAS可能具有相同的污染来源。风险评估结果表明，所有点位饮用水源水中的PFAS不会导致明显的生态风险；饮用水源水中全氟己烷磺酸（PFHxS）、全氟壬酸（PFNA）、六氟环氧丙烷二聚酸（HFPO-DA）和PFBS的水平符合美国国家主要饮用水法规对饮用水中PFAS的限量要求。鉴于传统及新型PFAS在饮用水源水中广泛检出，未来需开展持续跟踪监测活动，以保障居民饮用水安全。

## Abstract (en)

Per- and polyfluoroalkyl substances（PFAS） are notorious environmental contaminants that are often referred to as “forever chemicals”. Eastern China is a major region that produces and utilizes PFAS. These substances can enter the environment and migrate to drinking water sources， thereby posing potential ecological and health risks. To investigate the occurrence and potential risks of both legacy and novel PFAS in the drinking water sources of this region， a study was conducted on water samples collected from 13 drinking water sources. Anion exchange solid-phase extraction coupled with ultra performance liquid chromatography-triple quadrupole mass spectrometry（UPLC-MS/MS） was used to determine PFAS in water. The pollution levels and spatial distribution characteristics of 50 legacy and novel PFAS in these drinking water sources were studied in detail and their ecological and health risks were also assessed. Twenty-six types of PFAS were detected in the drinking water sources of the eastern region， with total mass concentrations ranging from 80.0 to 282 ng/L and a median mass concentration of 153 ng/L. The relatively high concentrations of PFAS in these drinking water samples is likely due to heavy industrial activities in this region. Nevertheless， the levels of perfluorooctanoic acid（PFOA） and perfluorooctanesulfonic acid（PFOS） in all sampling sites were far below the limits of national drinking water standards of China. The detected PFAS in drinking water sources were predominantly short-chain compounds， such as perfluorobutanoic acid（PFBA） and perfluorobutanesulfonic acid（PFBS）， which accounted for 27.6% and 20.8% of the total PFAS concentration， respectively. In contrast， long-chain PFAS（with more than nine carbons） constituted less than 2% of the total PFAS concentration. The observed higher proportions of short-chain PFAS are rationalized by their somewhat higher mobilities in aquatic environments. Low levels of various novel PFAS， such as hexafluoropropylene oxide dimer acid（HFPO-DA）， 1*H*，1*H*，2*H*，2*H*-perfluorooctanesulfonic acid（6∶2 FTS）， perfluoro（2-ethoxyethane）sulfonic acid（PFEESA）， 9-chlorohexadecafluoro-3-oxanonane-1-sulfonic acid（9Cl-PF3ONS）， and perfluorooctanesulfonamide（FOSA） were detected to varying degrees in the drinking water sources. This indicated that novel PFAS are being increasingly produced and used as alternatives to those restricted by international and Chinese regulations. The overall PFAS pollution levels and compositions in the drinking water sources of Eastern China are significantly influenced by nearby industrial activities， with wastewater discharged from chemical industrial parks and related enterprises contributing significantly to the elevated PFAS levels in the drinking water sources. Pearson correlation analysis suggested that the PFAS in these drinking water sources likely arise from common pollution sources. Ecological risk-assessment data reveal that the PFAS risk quotient in the abovementioned drinking water sources ranged from 9.7×10^-6^ to 8.9×10^-^³， which suggests that the PFAS levels at the sampling sites in this study evidently pose no ecological risks. The hazard indices for perfluorohexanoic acid（PFHxS）， perfluorononanoic acid（PFNA）， HFPO-DA， and PFBS in the examined drinking water sources ranged from 0.16 to 0.89. The detected levels of these four PFAS are compliant with the limits set by the national primary drinking water regulations of the United States. This study furnishes foundational data that provide a comprehensive understanding of the pollution profiles of PFAS in drinking water sources in Eastern China. Continuous monitoring is necessary to ensure drinking-water safety for residents given the widespread occurrence of both legacy and novel PFAS in drinking water.

全氟和多氟烷基物质（per-and polyfluoroalkyl substances，PFAS）是一类复杂的化合物，具有高热稳定性、低反应性及两疏性，在电镀、消防、电子和半导体、能源、建筑和施工、食品加工、制药、造纸及航空航天等领域具有重要应用^［1，2］^。另一方面，PFAS具有极强的持久性和迁移性^［3］^。PFAS的大量生产、广泛使用已经使其成为全球性污染物^［4］^。水体是全氟和多氟烷基物质重要的载体之一^［5］^，中国东部地区城市化水平高，工业产业密集，水体中的PFAS水平高于其他地区^［6］^。

PFAS具有蓄积性和广泛的生物毒性，一旦进入饮用水源水，将直接影响居民日常饮用水安全。相关研究表明，人类血液中PFAS的含量与饮用水中的含量具有显著正相关性^［7］^。PFAS进入人体后，将对健康造成不利影响。大量流行病学和毒理学研究表明PFAS暴露与多种不利效应有关，包括抑制儿童的免疫反应和神经发育^［8］^、干扰内分泌系统^［9］^、导致肝损伤，甚至诱发肾癌^［10］^等。因此，开展饮用水源水中PFAS污染调查对于摸清PFAS的污染水平，确保居民饮用水安全具有重要意义。

作为全球PFAS及相关化学品最主要的生产国和消费国，中国水环境中广泛检出PFAS^［11］^。Meng等^［12］^调查了东北大岭河干流和支流PFAS的污染水平，共检测到13种PFAS，检出水平为4.6~3410 ng/L，并发现工业废水排放显著影响当地地表水中PFAS含量。Ge等^［13］^调查了太湖流域饮用水源水中14种PFAS的污染情况，总含量为131.3~214.7 ng/L。Meng等^［14］^报道的北京密云水库中12种PFAS含量为5.30~8.50 ng/L，明显低于其他地区的饮用水源水中PFAS含量。近年来，受到对传统PFAS生产使用管控措施的影响，作为传统PFAS的替代品，新型PFAS产品不断涌现。但目前对这些新型PFAS在饮用水源水中的污染状况的认识极为匮乏。本研究以我国东部地区13处代表性饮用水源地为研究对象，通过分析饮用水源水中50种传统及新型PFAS的含量，获取了PFAS在饮用水源水中的污染水平及空间分布特征，并开展了初步的风险评估，以期为饮用水源水中PFAS的管控提供数据支持。

## 1 实验部分

### 1.1 仪器、试剂与材料

Triple Quad^TM^ 5500+液相色谱-三重四极杆质谱仪（美国SCIEX公司）；VM-B涡旋混合器、SBEQ-CG1824固相萃取装置（上海安谱实验科技股份有限公司）；MGS-2200氮气浓缩仪（上海爱朗仪器有限公司）；MS105DU/A分析天平（梅特勒托利多科技有限公司）；IQ7010超纯水设备（德国密理博有限公司）。

50种PFAS混合标准溶液（1~50 μg/mL）及9种同位素标记的PFAS内标混合溶液（2 μg/mL）均购自加拿大Wellington实验室，纯度均>98%。50种PFAS标准品包括：全氟丁酸（PFBA）、全氟戊酸（PFPeA）、全氟己酸（PFHxA）、全氟庚酸（PFHpA）、全氟辛酸（PFOA）、全氟壬酸（PFNA）、全氟癸酸（PFDA）、全氟十一烷酸（PFUnDA）、全氟十二烷酸（PFDoDA）、全氟十三烷酸（PFTrDA）、全氟十四烷酸（PFTeDA）、全氟十六烷酸（PFHxDA）、全氟十八烷酸（PFODA）、全氟丁烷磺酸钾（PFBS）、全氟戊烷磺酸钠（PFPeS）、全氟己烷磺酸钠（PFHxS）、全氟庚烷磺酸钠（PFHpS）、全氟辛烷磺酸钠（PFOS）、全氟壬烷磺酸钠（PFNS）、全氟癸烷磺酸钠（PFDS）、全氟十二烷磺酸钠（PFDoDS）、全氟-5-氧杂己酸（PF5OHxA）、全氟-5-氧杂戊酸（PF4OPeA）、全氟（2-乙氧基乙烷）磺酸钾（PFEESA）、全氟-3，6-二氧杂庚酸（3，6-OPFHpA）、9-氯十六氟-3-氧代壬烷-1-磺酸钾（9Cl-PF3ONS）、11-氯二十碳氟-3-氧十一烷-1-磺酸钾（11Cl-PF3OUdS）、4，8-二氧-3*H*-全氟壬酸钠（NaDONA）、六氟环氧丙烷二聚酸（HFPO-DA）、*N*-甲基全氟辛基磺酰胺（*N*-MeFOSA）、*N*-乙基全氟辛基磺酰胺（*N*-EtFOSA）、2-（*N*-甲基全氟-1-辛基磺酰胺基）-乙醇（*N*-MeFOSE）、2-（*N*-乙基全氟辛基磺酰胺基）乙醇（*N*-EtFOSE）、*N*-甲基全氟辛烷磺酰胺基乙酸（*N*-MeFOSAA）、*N*-乙基全氟辛烷磺酰胺乙酸（*N*-EtFOSAA）、全氟辛烷磺酰胺（FOSA）、1*H*，1*H*，2*H*，2*H*-全氟辛烷磺酸（4∶2FTS）、6∶2氟调聚物磺酸（6∶2FTS）、1*H*，1*H*，2*H*，2*H*-全氟十二烷磺酸（8∶2FTS）、2*H*，2*H*-全氟辛酸（6∶2FTCA）、2*H*，2*H*-全氟癸酸（8∶2FTCA）、2*H*，2*H*-全氟十二烷酸（10∶2FTCA）、1*H*，1*H*，2*H*，2*H*-全氟辛基磷酸二钠（6∶2PAP）、双（1*H*，1*H*，2*H*，2*H*-全氟辛基）磷酸钠（6∶2diPAP）、1*H*，1*H*，2*H*，2*H*-全氟癸基磷酸二钠（8∶2PAP）、双（1*H*，1*H*，2*H*，2*H*-全氟癸基）磷酸钠（8∶2diPAP）、（1*H*，1*H*，2*H*，2*H*-全氟辛基-1*H*，1*H*，2*H*，2*H*-全氟癸基）磷酸钠（6∶2/8∶2diPAP）、3-全氟丙基丙酸（FPrPA）、3-全氟戊基丙酸（FPePA）、3-全氟庚基丙酸（FHpPA）。9种同位素标记的PFAS内标包括：^13^C_4_-PFBA、^13^C_2_-PFHxA、^13^C_4_-PFOA、^13^C_5_-PFNA、^13^C_2_-PFDA、^13^C_2_-PFUnDA、^13^C_2_-PFDoDA、^18^O_2_-PFHxS和^13^C_4_-PFOS。

Oasis WAX固相萃取柱（6 mL，150 mg，60 μm）购自美国Waters公司。玻璃纤维滤膜（GF/F，0.7 μm）购自英国Whatman公司。实验中使用的甲醇（HPLC级）和乙腈（HPLC级）购自德国Merck公司。氨水（质量分数为25%，HPLC级）和乙酸铵（HPLC级）购自北京百灵威科技有限公司。

### 1.2 标准溶液的配制

将50种PFAS标准溶液用甲醇稀释成质量浓度为200~1 000 ng/mL的标准储备溶液。将9种PFAS内标混合溶液用甲醇稀释成200 ng/mL的混合内标稀释液。使用时，移取适量体积的标准储备溶液和混合内标稀释溶液进一步稀释成所需浓度的校准溶液。以上均于-30 ℃冰箱中冷藏保存。

### 1.3 样品采集

2023年7月，在东部地区选取13处饮用水源地开展样品采集工作，采样点地理位置见表1。使用1 L预清洁过的聚丙烯样品瓶在水下0.5 m处采集表层水样。采样前，使用水样荡洗采样瓶3次。采样完成后，将样品密封放置于4 ℃保温箱中运回实验室。

**表 1:** 饮用水源采样点的地理信息

Site	City	Longitude/°	Latitude/°
S1	Nanjing	118.5583	31.8688
S2	Nanjing	118.6974	32.0089
S3	Nanjing	118.8163	32.1512
S4	Lianyungang	119.1023	34.5869
S5	Lianyungang	119.0939	34.5332
S6	Taizhou	119.8822	32.2566
S7	Taixing	120.2700	31.9465
S8	Zhangjiagang	120.6093	31.98961
S9	Changshu	120.9052	31.7574
S10	Shanghai	121.1908	31.7624
S11	Shanghai	121.5758	31.4361
S12	Suzhou	120.2957	31.3157
S13	Suzhou	120.4904	31.0223

### 1.4 样品前处理

使用玻璃纤维滤膜过滤样品。取200 mL过滤后的水样，加入10 μL 200 ng/mL的内标混合溶液，混合均匀。将WAX固相萃取柱安装在固相萃取装置上，依次用6 mL 0.4%（v/v）氨水甲醇溶液、6 mL甲醇和6 mL纯水进行活化。将过滤后的水样通过聚丙烯大体积储存器转移至固相萃取柱，流速控制在约1 滴/s。上样结束后，使用6 mL 25 mmol/L乙酸铵水溶液润洗样品瓶，并将润洗液一并转移至固相萃取柱。再用0.25 mL甲醇淋洗固相萃取柱，弃去。待固相萃取柱完全干燥后，用10 mL 0.4%（v/v）氨水甲醇溶液洗脱，收集洗脱液。将洗脱液浓缩至近干，加入200 μL甲醇复溶，转移至聚丙烯进样瓶中，-18 ℃保存，待测。

### 1.5 仪器分析

采用Agilent RRHD C18 Eclipse Plus色谱柱（100 mm×2.1 mm，1.8 μm）分离目标物，进样量为5 μL，柱温设置为40 ℃。流动相A为2 mmol/L乙酸铵水溶液，B为乙腈，流速为0.3 mL/min。梯度洗脱程序：0~7.0 min，30%B~60%B；7.0~13.0 min，60%B~100%B；13.0~16.0 min，100%B；16.0~17.0 min，100%B~30%B；17.0~20.0 min，30%B。

离子源为电喷雾电离源（ESI），离子源温度为550 ℃，喷雾电压-4 500 V，气帘气设为240 kPa，碰撞气流速为9 L/min。50种目标物和9种内标的质谱参数见表2。

**表2:** 50种PFAS目标物和9种内标的质谱参数

Compound	Parent ions （*m/z*）	Product ions（*m/z*）	DP/V	CEs/eV	MDL/ （ng/L）	MQL/（ng/L）	IS
Heptafluorobutyric acid（PFBA）	212.8	168.9^*^	-40	-13	0.5	1.7	^13^C_4_-PFBA
Perfluoropentanoic acid（PFPeA）	263.0	219.0^*^/68.9	-40	-10/-50	0.2	0.5	^13^C_4_-PFBA
Perfluorohexanoic acid（PFHxA）	313.0	269.0^*^/118.9	-45	-13/-27	0.1	0.2	^13^C_2_-PFHxA
Perfluoroheptanoic acid（PFHpA）	363.1	319.0^*^/169.0	-30	-14/-24	0.1	0.3	^13^C_4_-PFOA
Perfluorooctanoic acid（PFOA）	413.0	369.0^*^/169.0	-40	-14/-24	0.1	0.3	^13^C_4_-PFOA
Perfluorononanoic acid（PFNA）	463.0	419.0^*^/169.0	-35	-16/-24	0.1	0.3	^13^C_5_-PFNA
Perfluorodecanoic acid（PFDA）	512.9	469.0^*^/219.0	-40	-18/-26	0.1	0.4	^13^C_2_-PFDA
Perfluoroundecanoic acid（PFUnDA）	563.1	519.0^*^/319.0	-70	-16/-28	0.1	0.3	^13^C_2_-PFUnDA
Perfluorododecanoic acid（PFDoDA）	613.1	569.0^*^/169.0	-70	-18/-36	0.1	0.4	^13^C_2_-PFDoDA
Perfluorotridecanoic acid（PFTrDA）	663.0	619.0^*^/168.9	-65	-20/-38	0.1	0.3	^13^C_2_-PFDoDA
Perfluorotetradecanoic acid（PFTeDA）	713.1	669.0^*^/168.9	-85	-20/-38	0.1	0.5	^13^C_2_-PFDoDA
Perfluorohexadecanoic acid（PFHxDA）	813.0	769.0^*^/168.9	-90	-18/-30	0.3	1.0	^13^C_2_-PFDoDA
Perfluorooctadecanoic acid（PFODA）	913.1	869.0^*^/168.9	-40	-25/-45	0.3	0.8	^13^C_2_-PFDoDA
Perfluorobutanesulfonate（PFBS）	298.7	79.9^*^/98.9	-90	-70/-38	0.1	0.3	^18^O_2_-PFHxS
Potassium perfluoropentanesulfonate（PFPeS）	349.1	79.9^*^/98.9	-90	-80/-50	0.1	0.2	^18^O_2_-PFHxS
Sodium perfluorohexanesulfonate（PEHxS）	398.7	79.9^*^/98.9	-90	-90/-72	0.1	0.3	^18^O_2_-PFHxS
Sodium perfluoroheptanesulfonate（PFHpS）	449.0	79.9^*^/98.9	-95	-100/-80	0.1	0.2	^13^C_4_-PFOS
Sodium perfluorooctanesulfonate（PFOS）	498.9	79.9^*^/98.9	-105	-110/-98	0.1	0.3	^13^C_4_-PFOS
Sodium perfluorononanesulfonate（PFNS）	548.8	79.9^*^/98.9	-110	-115/-105	0.1	0.2	^13^C_4_-PFOS
Sodium perfluorodecanesulfonate（PFDS）	599.0	79.9^*^/98.9	-120	-124/-110	0.1	0.2	^13^C_4_-PFOS
Sodium perfluorododecanesulfonate（PFDoDS）	699.1	79.9^*^/98.9	-130	-135/-120	0.1	0.2	^13^C_4_-PFOS
Perfluoro-5-oxahexanoic acid（PF5OHxA）	279.0	85.0^*^/235.0	-35	-17/-9	0.1	0.5	^13^C_2_-PFHxA
Perfluoro-4-oxapentanoic acid（PF4OPeA）	229.0	85.0^*^/185.0	-35	-24/-9	0.1	0.4	^13^C_2_-PFBA
Potassium perfluoro（2-ethoxyethane）sulfonate（PFEESA）	314.8	134.9^*^/82.9	-45	-30/-26	0.2	0.5	^13^C_2_-PFHxA
Perfluoro-3，6-dioxaheptanoic acid（3，6-OPFHpA）	295.0	201.0^*^/85.0	-25	-11/-33	0.3	0.9	^13^C_2_-PFHxA
Potassium 9-chlorohexadecafluoro-3-oxanonane-1-sulfonate（9Cl-PF3ONS）	530.8	351.0^*^/83.0	-50	-38/-60	0.1	0.5	^13^C_4_-PFOS
Potassium 11-chloroeicosafluoro-3-oxaundecane-1-sulfonate（11Cl-PF3OUdS）	630.9	450.9^*^/83.0	-35	-39/-91	0.1	0.3	^13^C_4_-PFOS
Sodium dodecafluoro-3*H*-4，8-dioxanonanoate（NaDONA）	376.9	250.9^*^/84.8	-35	-16/-40	0.2	0.5	^13^C_4_-PFOA
Hexafluoropropylene oxide dimer acid（HFPO-DA）	329.0	168.9^*^/184.9	-10	-18/-33	0.1	0.3	^13^C_2_-PFHxA
*N*-Methylperfluorooctane sulfonamide（*N*-MeFOSA）	511.9	169.0^*^/219.0	-142	-36/-33	0.2	0.6	^13^C_2_-PFDoDA
*N*-Ethylperfluorooctane sulfonamide（*N*-EtFOSA）	526.0	169.0^*^/219.0	-120	-37/-36	0.1	0.4	^13^C_2_-PFDoDA
2-（*N*-Methyl perfluorooctanesulfonamido）ethanol（*N*-MeFOSE）	616.1/602.0	58.9^*^/45.0	-80	-74/-51	0.6	1.9	^13^C_2_-PFDoDA
2-（*N*-Ethyl perfluorooctanesulfonamido）ethanol（*N*-EtFOSE）	630.0/616.1	58.9^*^/45.0	-60	-60/-60	0.6	2.0	^13^C_2_-PFDoDA
*N*-Methylperfluorooctanesulfonamidoacetic acid（*N*-MeFOSAA）	570.1	419.0^*^/219.0	-85	-27/-36	0.1	0.4	^13^C_4_-PFOS
*N*-Ethylperfluorooctanesulfonamidoacetic acid（*N*-EtFOSAA）	584.2	419.0^*^/219.0	-80	-28/-35	0.1	0.5	^13^C_4_-PFOS
Perfluorooctanesulfonamide（FOSA）	498.1	77.9^*^/478.0	-76	-98/-35	0.1	0.2	^13^C_4_-PFOS
Sodium 1*H*，1*H*，2*H*，2*H*-perfluorohexanesulfonate（4∶2FTS）	327.1	307.0^*^/80.9	-47	-28/-55	0.3	1.1	^18^O_2_-PFHxS
Sodium 1*H*，1*H*，2*H*，2*H*-perfluorooctanesulfonate（6∶2FTS）	427.1	407.0^*^/80.9	-50	-33/-36	0.3	1.1	^18^O_2_-PFHxS
Sodium 1*H*，1*H*，2*H*，2*H*-perfluorodecanesulfonate（8∶2FTS）	527.1	507.0^*^/80.9	-80	-36/-89	0.2	0.7	^18^O_2_-PFHxS
2*H*，2*H*-Perfluorooctanoic acid（6∶2FTCA）	377.1	292.9^*^/62.9	-54	-19/-10	0.3	1.0	^13^C_2_-PFHxA
2*H*，2*H*-Perfluorodecanoic acid（8∶2FTCA）	477.1	392.9^*^/62.9	-64	-19/-10	0.3	1.0	^13^C_4_-PFOA
2*H*，2*H*-Perfluorododecanoic acid（10∶2FTCA）	577.1	492.9^*^/62.9	-80	-23/-43	0.6	2.0	^13^C_4_-PFOA
Sodium 1*H*，1*H*，2*H*，2*H*-perfluorooctyl phosphate（6∶2PAP）	443.0	79.1^*^/96.9	-32	-100/-20	0.7	2.3	^13^C_2_-PFHxA
Sodium bis（1*H*，1*H*，2*H*，2*H*-perfluorooctyl）phosphate（6∶2diPAP）	789.0	79.1^*^/96.8	-25	-168/-81	0.1	0.4	^13^C_2_-PFDoDA
Sodium 1*H*，1*H*，2*H*，2*H*-perfluorodecylphosphate（8∶2PAP）	543	79.1^*^/96.9	-81	-127/-20	0.3	0.9	^13^C_4_-PFOA
Sodium bis（1*H*，1*H*，2*H*，2*H*-perfluorodecyl）phosphate（8∶2diPAP）	989.0	79.1^*^/542.8	-150	-173/-32	0.7	2.3	^13^C_2_-PFDoDA
Sodium（1*H*，1*H*，2*H*，2*H*-perfluorooctyl-1*H*，1*H*，2*H*，2*H*-perfluorodecyl）phosphate（6∶2/8∶2diPAP）	889.0	79.1^*^/442.9	-80	-160/-30	0.4	1.3	^13^C_2_-PFDoDA
3-Perfluoropropyl propanoic acid（FPrPA）	241.0	177.0^*^/62.9	-13	-11/-10	0.3	1.2	^13^C_2_-PFHxA
3-Perfluoropentyl propanoic acid（FPePA）	341.0	237.0^*^/62.9	-20	-19/-13	0.3	1.1	^13^C_2_-PFHxA
3-Perfluoroheptyl propanoic acid（FHpPA）	441.0	337.0^*^/317.0	-44	-17/-27	0.5	1.5	^13^C_4_-PFOA
Perfluoro-*n*-（^13^C_4_）butanoic acid（^13^C_4_-PFBA）	216.8	171.9	-50	-12			
Perfluoro-*n*-（1，2-^13^C_2_）hexanoic acid（^13^C_2_-PFHxA）	315.0	270.0	-55	-14			
Perfluoro-*n*-（1，2，3，4-^13^C_4_）octanoic acid（^13^C_4_-PFOA）	417.0	372.0	-70	-20			
Perfluoro-*n*-（1，2，3，4，5-^13^C_5_）nonanoic acid（^13^C_5_-PFNA）	468.0	423.0	-70	-22			
Perfluoro-*n-*（1，2-^13^C_2_）decanoic acid（^13^C_2_-PFDA）	514.9	470.0	-75	-17			
Perfluoro-*n*-（1，2-^13^C_2_）undecanoic acid（^13^C_2_-PFUnDA）	565.1	520.0	-60	-15			
Perfluoro-*n*-（1，2-^13^C_2_）dodecanoic acid（^13^C_2_-PFDoDA）	615.1	570.0	-60	-15			
Sodium perfluoro-1-hexane（^18^O_2_）sulfonate（^18^O_2_-PFHxS）	402.8	102.9	-90	-75			
Sodium perfluoro-1-（1，2，3，4-^13^C_4_）octanesulfonate（^13^C_4_-PFSA）	502.9	79.9	-90	-95			

DP： declustering potential； CEs： collision energies； MDL： method detection limit； MQL： method quantification limit； IS： internal standard； * quantitative ion.

### 1.6 质量保证与质量控制

分析过程中与样品直接接触的容器均为聚丙烯塑料制品。样品处理过程中添加全程空白及实验室空白样品，并按照与实际样品相同的流程进行前处理和仪器测试。除PFBA 和PFOA在空白样品中有痕量检出（<0.3 ng/L）外，其他目标物均未检出。样品进行仪器分析之前用流动相清洗色谱系统30 min以上。测得含量低于方法检出限的物质认为其含量为0，低于方法定量限但高于检出限的物质按检出水平计算。

### 1.7 风险评估

采用风险熵值法（RQ）开展水生态风险评估^［15］^。当RQ≥1时，表明极有可能产生风险，需给予关注并采取适当措施以降低风险；当0.1≤RQ<1时，表明环境处于安全水平，但应长期观察避免后续产生风险；当RQ<0.1时，表明环境是安全的^［16］^。RQ计算公式如下：

RQ=MEC/PNEC（1）

式中，MEC为目标物监测质量浓度（ng/L），PNEC为预测无效应浓度（ng/L），该值参考Zhang等^［17］^、Riaz等^［18］^的研究以及NORMAN毒理学数据库中的最低预测无效应浓度的数据^［19］^。

鉴于PFAS在水处理厂的去除效果不佳^［20，21］^，饮用水源水中的PFAS极可能在自来水厂处理过程中被保留下来从而进入末梢水中并进一步通过饮水进入人体。为了评估居民通过饮水摄入PFAS产生的健康风险，参考美国环保署（US EPA）于 2024 年 4 月 26 日发布的最终版的国家主要饮用水法规（NPDWR）^［22］^，采用危害指数（HI）开展健康风险评估。该评估方法考虑了PFNA、HFPO-DA、PFHxS和PFBS的联合健康效应。当HI>1时，表明饮用水中PFAS存在健康风险。当HI≤1时，表明不存在明显的健康风险。HI计算公式如下：


$$HI=\frac{C_{HFPO-DA}}{10\;ng/L}+\frac{C_{PFBS}}{2000\;ng/L}+\frac{C_{PFNA}}{10\;ng/L}+\frac{C_{PFHxS}}{10\;ng/L}$$ （2）

式中，*C*_HFPO-DA_、*C*_PFBS_、*C*_PFNA_和*C*_PFHxS_分别代表HFPO-DA、PFBS、PFNA和PFHxS的含量（ng/L）。

## 2 结果与讨论

### 2.1 线性关系、检出限和回收率

配制系列浓度的50种PFAS混合标准溶液，以待测组分与内标浓度的比值为横坐标，以待测组分与内标物峰面积的比值为纵坐标，绘制校准曲线。50种PFAS在各自范围内线性关系良好，相关系数均大于0.990。以水作为空白基质，并添加痕量目标物，采用与实际水样相同的前处理与仪器分析方法进行分析。方法检出限和定量限分别定义为7个痕量加标样品测定含量的3倍和10倍标准偏差。对于50种PFAS，方法检出限为0.1~0.7 ng/L，定量限为0.2~2.3 ng/L。向200 mL水中添加0.2~2 ng的目标物，重复测定6次，加标回收率为64%~149%，相对标准偏差<20%，其中仅有10∶2FTCA、PFHxDA、PFODA和FPrPA 4种物质的RSD为10%~20%，其他46种PFAS均低于10%。

### 2.2 东部地区饮用水源水中传统和新型PFAS的赋存状况

我国东部地区饮用水源水中共检出26种全氟和多氟烷基物质，总含量水平（Σ_26_PFAS）为80.0~282 ng/L，中位数值为153 ng/L，平均含量为175 ng/L。其中，PFBA、PFHxA、PFHpA、PFOA、PFNA、PFBS、PFHxS、PFOS、HFPO-DA和6∶2FTS 10种PFAS的检出率为100%，其他目标物检出率均低于40%（见表3）。东部地区饮用水源水中检出的PFAS以短链的类似物为主（图1），其中PFBS、PFBA和PFHxA分别占总含量的27.6%、20.8%和13.6%；而长链全氟和多氟烷基物质（碳链长度>9）的占比低于2%。Zhang等^［23］^报道的2018-2020年采集的长江江苏段饮用水源水中PFAS以PFOA为主，平均占比达34.4%。但本研究发现短链的PFBS和PFBA已经成为东部地区饮用水源水中最主要的PFAS，这与Zhu等^［24］^和Wang等^［25］^的研究结果相似，说明在国家对PFOA实施严格管控后，水环境中PFOA已有下降趋势。而短链的PFAS由于体积小、水溶性大、吸附性差，导致其在水环境中迁移能力更强^［26］^，更易进入饮用水源水中。另一方面，作为传统PFAS替代品之一，短链PFAS越来越多地被投入生产和应用，也导致其在饮用水源水中检出率和检出水平升高。

**表 3:** 东部地区饮用水源水中PFAS的检出情况

Substance	DF/%	Contents/（ng/L）			
		Min	Max	Median	Mean
PFBA	100	20.7	55.5	32.8	34.7
PFPeA	31	n.d.	35.1	n.d.	7.09
PFHxA	100	4.28	67.3	20.5	25.3
PFHpA	100	0.80	4.50	1.80	2.32
PFOA	100	4.33	56.4	20.7	23.9
PFNA	100	0.55	1.78	1.08	1.09
PFDA	23	n.d.	4.64	n.d.	0.41
PFUndA	15	n.d.	16.2	n.d.	1.26
PFDoDA	8	n.d.	43.9	n.d.	3.37
PFTrDA	8	n.d.	21.6	n.d.	1.66
PFTeDA	23	n.d.	4.13	n.d.	0.34
PFBS	100	3.60	94.2	59.0	46.0
PFHxS	100	0.26	5.58	1.25	1.89
PFOS	100	0.84	5.05	1.54	2.31
PFDS	8	n.d.	44.4	n.d.	3.41
PFNS	8	n.d.	15.9	n.d.	1.22
PFEESA	38	n.d.	0.43	n.d.	0.11
9Cl-PF3ONS	31	n.d.	0.31	n.d.	0.08
HFPO-DA	100	0.43	3.82	1.27	1.41
NMeFOSA	8	n.d.	0.13	n.d.	0.01
*N*-MeFOSE	38	n.d.	3.07	n.d.	1.15
*N*-EtFOSE	38	n.d.	1.57	n.d.	0.55
FOSA	23	n.d.	0.41	n.d.	0.07
6∶2FTS	100	1.43	70.3	2.35	15.3
8∶2FTCA	31	n.d.	2.32	n.d.	0.36
8∶2diPAP	15	n.d.	0.23	n.d.	0.04

Contents were corrected for the salt content. DF： detection frequency； n.d.： not detected.

**图1:**
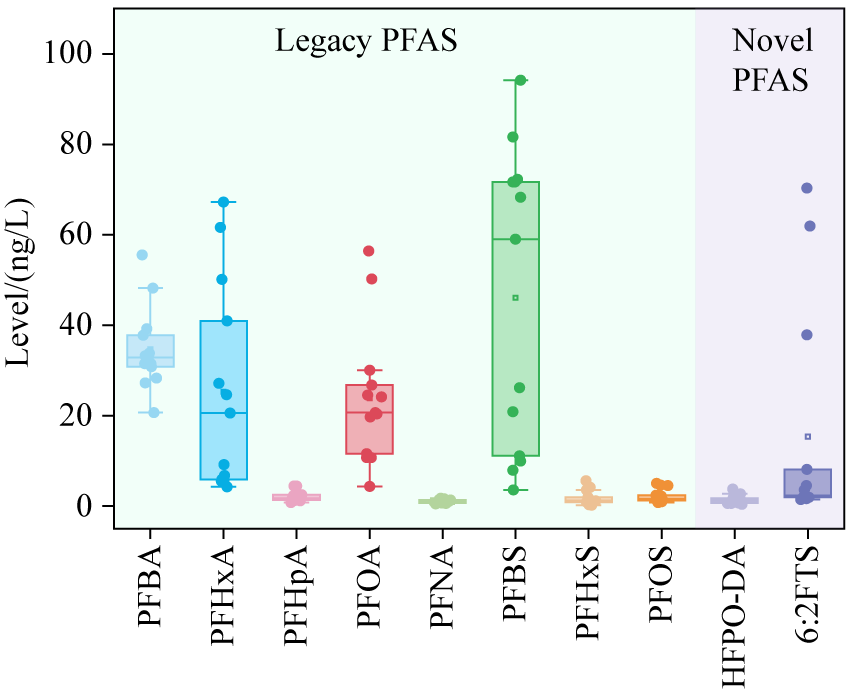
东部地区饮用水源水中高检出PFAS的含量水平（*n*=13）

目前，我国生活饮用水卫生标准（GB 5749-2022）仅规定了生活饮用水中PFOA和PFOS的限量标准，分别为80 ng/L和40 ng/L；而对生活饮用水地表水源水质的管理则为符合地表水环境质量标准（GB 3838-2002）要求。由于我国现行的地表水环境质量标准（GB 3838-2002）对集中式生活饮用水地表水源地的水质评价项目缺乏对全氟化合物的限量值，因此本实验将所测的地表水源水中PFAS的含量与生活饮用水的限量标准作比较。本研究中东部地区饮用水源水中PFOA和PFOS的含量均低于我国生活饮用水卫生标准（GB 5749-2022）中的限量标准。但与近年来报道的其他地区饮用水源水中PFAS水平相比（见表4），如柳思帆^［27］^对北京密云水库的研究，Cao等^［28］^对天津玉桥水库的研究，本研究中东部地区饮用水源水中PFAS的检出水平相对较高，可能与东部地区化工园区数量多、工业生产活动频繁有关^［29-32］^。

**表 4:** 不同地区饮用水源水中PFAS的含量比较

Area	Contents/（ng/L）							Ref.
	PFBA	PFHxA	PFOA	PFBS	PFOS	Novel PFAS	Others	
Jingsu section of the Yangtze River	0.87-7.9	0.20-91	3.9-45	0.50-15	0.06-4.9	/	<0.01-5.2	［^23^］
Songhua River	/	n.d.-0.21	<0.12-0.69	/	0.04-0.27	/	n.d.-0.23	［^24^］
Liao River	n.d.-99	1.5-13	n.d.-28	0.95-13	n.d.-6.6	/	n.d.-74	［^24^］
Yellow River	1.6-54	0.37-47	2.0-42	n.d.-6. 7	0.86-41	/	n.d.-101	［^24^］
Miyun Reservoir	1.5-4.3	n.d.-0.71	n.d.-2.8	n.d.	n.d.-0.72	/	n.d.-0.97	［^27^］
Guanting Reservoir	3.0-49	n.d.-4.5	15-71	n.d.-0.48	n.d.-1.9	/	n.d.-19	［^27^］
Yuqiao Reservoir	2.8-67	0.34-25	0.48-5.3	0.09-4.2	n.d.-5.5	/	n.d.-6.9	［^28^］
Netherland	2.5-15	1.2-6.2	2.4-42	0.09-19	0.46-3.1	n.d.-19	n.d.-20	［^21^］
Eastern China	21-56	4.3-67	4.3-57	3.6-94	0.84-5.1	n.d.-70	n.d.-44	this study

Novel PFAS included HFPO-DA， 6∶2FTS， PFEESA， 9Cl-PF3ONS， FOSA， etc.； /： no data.

尽管传统的PFOA、PFOS、PFHxS已被列入我国重点管控新污染物清单，但其在饮用水源水中仍能被100%检出，这可能与这些物质在部分应用领域（如半导体、医疗、能源领域等）具有豁免权，目前仍在继续使用有关。此外，含有这些PFAS的历史遗留产品也在不断向环境释放。在本次针对饮用水源水的调查中，新型PFAS，如HFPO-DA、6∶2FTS、PFEESA、9Cl-PF3ONS、FOSA等也有不同程度检出，说明受到国际社会和中国政府对传统全氟类化合物管控的影响，新型全氟和多氟烷基物质正作为替代品逐渐被企业采用以满足生产需求。

### 2.3 东部地区饮用水源水中PFAS的空间分布特征

为了研究PFAS在东部地区饮用水源水中的空间分布特征，选取检出率为100%的10种PFAS进行分析，其在不同点位饮用水源水中的含量和组成如图2所示。其中，S9点位饮用水源水中PFAS的检出水平最高，为271 ng/L。该采样点位于常熟，临近我国著名的有机氟化学工业园，饮用水源水中较高的PFAS水平可能与园区工业废水的排放有关^［33］^。大多数点位饮用水源水中短链的PFBS和PFBA占比较大，两者约占10种PFAS总量的54%，但采集自连云港（S4和S5）和泰州（S6）点位饮用水源水中新型的6∶2FTS的占比明显高于其他地区（28%~51%）。作为传统PFOS的替代品，6∶2FTS在工业生产中有广泛应用^［1］^。饮用水源水中较高的6∶2FTS检出水平可能是传统PFAS产品的替代升级导致。采集自太湖东北部S12点位饮用水源水中PFAS的含量相对较高（184 ng/L）。该点位较高的检出浓度可能与“引江济太”工程密切相关^［34］^。之前研究发现，太湖北部河流输入显著影响太湖水体中PFAS的水平^［33］^。PFAS在南京（S1~S3）及上海（S10和S11）采样点位饮用水源水中的水平相对均衡，个别点位含量略有升高可能与水环境中PFAS形成的面源污染有关^［35］^。总体来看，东部地区饮用水源水中PFAS污染水平受到采样点周边工业生产活动影响较大。

**图2:**
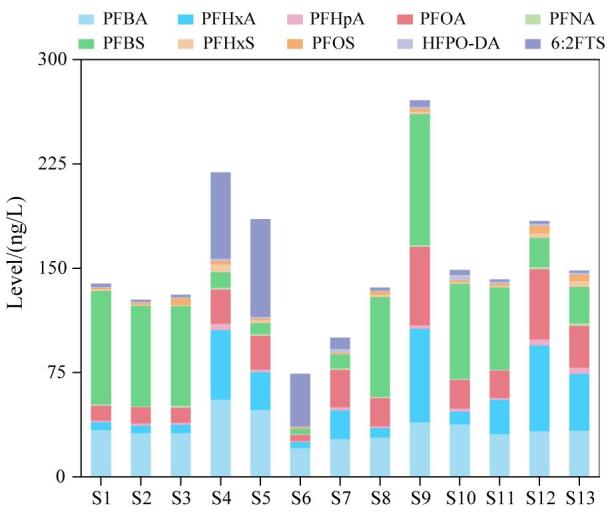
东部地区饮用水源水中PFAS的空间分布情况

### 2.4 PFAS各物质间的相关性分析

为了进一步研究饮用水源水中PFAS的潜在污染来源，对检出率为100%的化合物开展皮尔森相关性分析。为纠正偏态分布，数据经过了以10为底的对数变换，结果如图3所示。饮用水源水中PFHxA和PFOA具有显著正相关关系（*r*=0.91，*p*<0.001），说明二者可能来自相同污染源，这与二者在应用功能的相似性及应用领域的高度重合密切相关。除了污染源，化合物的环境行为、前体化合物的降解转化等过程也会影响到样品中目标物质含量的相关性。在满足*p*<0.01级别（双尾）条件下表现出正相关的还有PFHpA和PFHxA（*r*=0.77）、PFNA和PFHpA（*r*=0.75）、PFHxS和PFHxA（*r*=0.69）、PFHxS和PFHpA（*r*=0.88）、PFHxS和PFNA（*r*=0.71）及PFOS和PFNA（*r*=0.76）。这与Ahrens等^［36］^报道的同类别PFAS具有相同来源的结论一致。

**图3:**
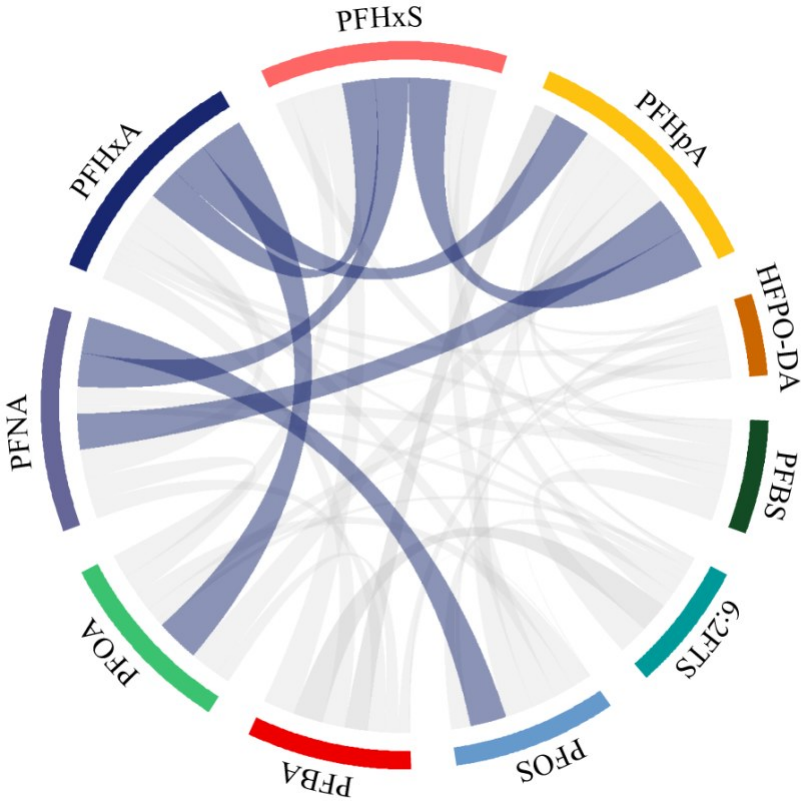
PFAS之间的相关性

### 2.5 饮用水源水风险评估

对饮用水源水中检出的26种PFAS开展生态风险评估。计算结果表明，东部地区饮用水源水中PFAS产生的风险熵为9.7×10^-6^~8.9×10^-3^。图4a展示了检出率为100%的10种PFAS的风险熵值。所有物质风险熵值均低于0.01，表明饮用水源水中出现的全氟化合物未对当地水生态系统产生风险。根据NPDWR^［22］^中对于PFAS危害指数的计算方法，东部地区饮用水源水中PFHxS、PFNA、HFPO-DA和PFBS的危害指数为0.16~0.89（图4b）。所有点位饮用水源水中这4种PFAS的水平均符合美国国家主要饮用水法规^［22］^对饮用水中全氟化合物的限量要求。

**图4:**
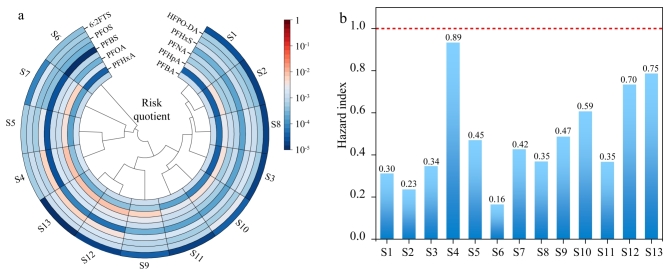
饮用水源水中PFAS的（a）风险熵值和（b）危害指数

## 3 结论

受到国际社会和国内政府对传统PFAS管控的影响，新型PFAS作为传统PFOA、PFOS等的替代品逐渐被生产和使用。尽管其检出水平不高，但在东部地区饮用水源水中已发现多种新型PFAS。短链的PFBA和PFBS由于具有更强的迁移性，已成为现阶段东部地区饮用水源水中占比最大的PFAS。东部地区饮用水源水中PFAS检出水平受到采样点周边工业生产活动影响较大，但PFOA和PFOS水平远低于我国生活饮用水卫生标准中的限值。风险评估结果表明饮用水源水中出现的全氟化合物不会对水生态系统造成风险，且所有点位饮用水源水中PFHxS、PFNA、HFPO-DA和PFBS的水平不会产生明显健康风险。本工作为全面了解我国饮用水源水中PFAS的污染状况提供了数据基础。鉴于传统及新型PFAS在饮用水源水中广泛检出，未来需开展持续跟踪监测活动，以保障居民饮用水安全。
